# Women’s voice on changes in childbirth care practices: a qualitative approach to women’s experiences in Brazilian private hospitals participating in the Adequate Childbirth Project

**DOI:** 10.1186/s12978-022-01539-y

**Published:** 2023-01-24

**Authors:** Andreza Pereira Rodrigues, Débora Cecília Chaves de Oliveira, Maysa Luduvice Gomes, Lucia Regina de Azevedo Nicida, Jacqueline Alves Torres, Amanda da Trindade Dias Coutinho, Beatriz da Silva Soares de Souza Cravo, Juliana Guimarães Dantas, Thays Basílio Oliveira, Rosa Maria Soares Madeira Domingues

**Affiliations:** 1 Escola de Enfermagem Anna Nery, Universidade Federal do Rio de Janeiro (UFRJ), Rua Afonso Cavalcanti, 275. Cidade Nova, Rio de Janeiro, RJ CEP: 20211-130 Brazil; 2 Faculty of Nursing, Rio de Janeiro State University (UERJ), Rio de Janeiro, RJ Brazil; 3 Casa de Oswaldo Cruz (COC), Oswaldo Cruz Foundation (Fiocruz), Rio de Janeiro, RJ Brazil; 4 Institute for Health Care Improvement, Rio de Janeiro, Brazil; 5 Laboratory of Clinical Research in STD/AIDS, Evandro Chagas National Institute of Infectology, Oswaldo Cruz Foundation (Fiocruz), Rio de Janeiro, RJ Brazil

**Keywords:** Women’s perspective, Hospitals, Private, Birth, Cesarean section, Quality improvement, Change, Organizational

## Abstract

**Background:**

In Brazil, childbirth practices are strongly marked by surgical events and particularly in the private sector cesarean sections reach rates above 80%. The National Supplementary Health Agency proposed the Adequate Childbirth Project (PPA), a quality improvement project developed at Brazilian hospitals with the aim of changing the current model of childbirth care and reducing unnecessary cesarean sections. The objective of this study is to assess how the participation of women in the process of improving quality childbirth care occurred in two hospitals participating in the PPA.

**Method:**

Qualitative study, based on interviews with 102 women attended at two hospitals that took part in the first and second stages of the “Healthy Birth”, an evaluative hospital-based research, conducted in 2017–2018, that assessed the degree of implementation and the effects of PPA. After thematic content analysis, supported by MaxQda software, three categories emerged: (1) how women gathered knowledge about the PPA, (2) how women perceived it, and (3) which are their suggestions for the PPA improvement.

**Results:**

The PPA was unknown to most women before delivery. A polysemy of terms, including adequate childbirth, promotes recognition of the “new” model of care. Visits to the maternity hospital and antenatal care groups for pregnant women are opportunities for contacts that change the perception of what childbirth can be. Women have expectations of a relationship with maternity that is not limited to the moment of delivery. The listening channels established between hospitals and women are fragile and not systematized. By increasing the supply of listening spaces, one can also increase the request to leave their suggestions and contributions, and thus gain more allies in improving the project. Women are not yet included as PPA agents and their voices are silenced.

**Conclusions:**

Women’s participation to improve childbirth care is relevant and necessary. The women’s voice in the PPA is still incipient, and maternity hospitals and health plan operators should create strategies to insert and engage them. Women’s voices should be listened to not only during but also before and after childbirth.

## Plain Language Summary

In Brazil, childbirth practices are strongly marked by surgical events and particularly in the private sector cesarean sections reach rates above 80%. The Adequate Childbirth Project (PPA) is a quality improvement project developed at Brazilian hospitals with the aim of changing the current model of childbirth care and reducing unnecessary cesarean sections. A qualitative study was developed in order to understand how hospitals have included the participation of women in the PPA. Based on interviews with 102 women, the present study shows that the PPA was unknown to most women before delivery. A polysemy of terms, including adequate childbirth, promotes recognition of the “new” model of care. Visits to the hospital and antenatal care groups for pregnant women are opportunities for contacts that change the perception of what childbirth can be. Women’s participation to improve childbirth care is relevant and necessary. The women’s voice in the PPA is still incipient and women are not yet included as agents of change, and their voices are silenced. Hospitals and health plan operators should create strategies to engage them. Women’s voices may be listened to not only during but also before and after childbirth.

## Background

In Brazil, childbirth practices are strongly marked by surgical events and excessive interventions during labor, which have been analyzed as disrespect and abuse, and, sometimes, as obstetric violence [1]. There is currently no evidence of benefits from cesarean sections (CS) rates greater than 10–15% [2]. Therefore, the CS rate above 50% in Brazil mobilized several actions. In a health system with very distinct characteristics between public and private subsectors [3], and with cesarean rates around 40% and 80%, respectively, the private sector was the target of a complaint to the Public Ministry regarding childbirth practices [4].

In response, the National Supplementary Health Agency (Agência Nacional de Saúde Suplementar—ANS) proposed the “Adequate Childbirth Project” (Projeto Parto Adequado—PPA), a quality improvement project developed at Brazilian hospitals with the aim of changing the current model of childbirth care and reducing unnecessary cesarean sections [5, 6]. The PPA is structured in three phases, as follows: Phase 1, developed in 2015 and 2016, aimed to test the intervention and was joined by 35 public and private hospitals and 19 health plan operators. Phase 2, beginning in 2017 and still ongoing, is characterized by extending the project to a variety of healthcare providers and carriers. Finally, Phase 3, launched in October 2019, aims to disseminate the strategies for improving the quality of delivery and birth care on a large scale, with the possibility of including the set of maternity hospitals and operators in Brazil. In 2021, the National Health Council, the highest level of social control in the Brazilian Unified Health System recommended the continuity and improvement of this project due to its importance for the qualification of obstetric care [7].

The PPA quality improvement project is composed of four driving factors: governance, women’s participation, reorganization of the childbirth model of care, and monitoring of indicators. In each driver, several activities were defined to be implemented by the hospitals that joined the program. The driver “participation of women and families” seeks to increase the “empowerment of women and families so they actively participate in the entire process of pregnancy, birth and postpartum care”. Therefore, what is expected is the empowerment of women in their own childbirth process, and this is achieved by improving the forms and channels of communication between maternity hospitals and women and by engaging women in the improvement of childbirth care in each maternity hospital. Women’s participation in changing models of obstetric care has become increasingly relevant [8–13], i.e., changes that exclude women’s voices may be doomed to failure in the medium- and long-term.

This article aims to assess how the participation of women in the process of improving quality childbirth care occurred in two private hospitals that participated in the Adequate Childbirth Project.

## Methods

An evaluative survey of the first phase of the Adequate Childbirth Project was conducted by the Sérgio Arouca National School of Public Health (ENSP), of Oswaldo Cruz Foundation (Fiocruz). This research was entitled “Healthy Birth” and used a mixed methods approach, with a cross-sectional design in the quantitative component [4]. Data were collected in two moments: Moment 1 (M1)—that aimed to assess the degree of implementation of PPA, and Moment 2 (M2)—that aimed to assess the sustainability of PPA.

Moment 1 of the quantitative component took place from March 2017 to August 2017, covering twelve of the 23 private hospitals that participated in the first phase of the PPA. Inclusion criteria were: hospital location (according to Brazilian regions); type of hospital (hospitals owned or not owned by health plan operators); and hospital performance (hospital performance was classified as “good” or “bad”, evaluated by the PPA coordination). In each maternity hospital, a sample size of approximately 400 women was calculated, aiming to detect a 10% reduction in the proportion of cesarean sections. All puerperal women with a hospital delivery of a live birth, of any gestational age or birth weight, or of a stillbirth, with gestational age ≥ 22 weeks or weight ≥ 500 g, were considered eligible for the study. Women with hearing impairment, foreigners who did not speak Portuguese, women with pregnancies with three or more fetuses, and women hospitalized for judicial termination of pregnancy were considered ineligible. In each hospital, women were invited to participate in the study consecutively until the planned sample was reached. Moment two of the quantitative component took place from May 2018 to August 2018, with only 8 of the 12 initial hospitals. Four hospitals were excluded due to geographic location and similar results. In each of these eight hospitals, the methodological procedures of M1 were repeated, with interviews with approximately 400 women in each hospital and extraction of data from women’s and newborns’ records [4].

The qualitative component in M1 took place from July 2017 to August 2018, in 8 of the 12 hospitals that showed the best results under the degree of implementation, and the qualitative component in M2 took place from September 2018 to November 2019, in four of the eight hospitals with the best implementation performance. The timeline of the Adequate Childbirth Project and “Healthy Birth” study are presented in Gomes et al. [14].

In both M1 and M2, qualitative data collection included hospital managers, health professionals directly involved in childbirth (doctors and nurses), non-participant observation and, finally, telephone contact with the women approximately 6 months after childbirth, for an in-depth interview. More details about data collection, including instruments developed for this study, contextual aspects and protocols established by the Healthy Birth research can be found in Torres et al. [4] and Domingues et al. [15].

**Fig. 1:**
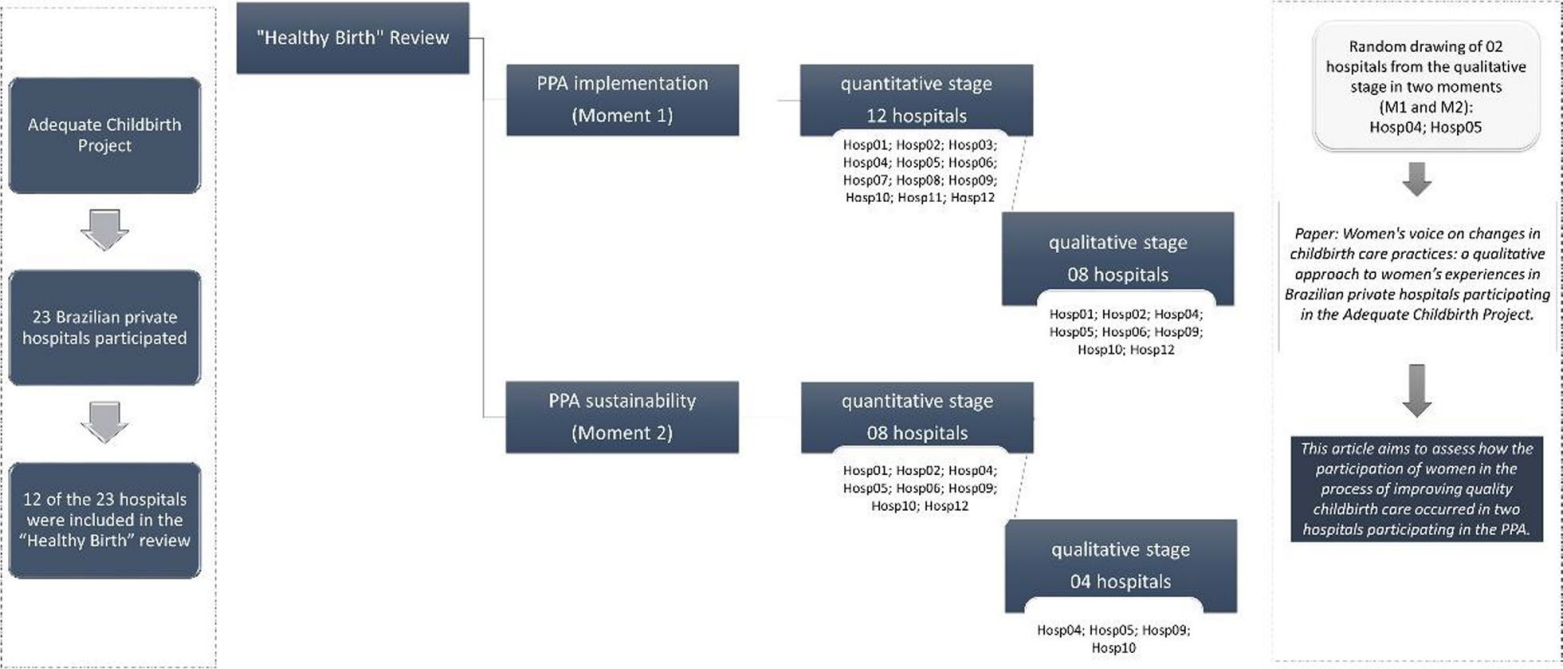
Selection process of hospitals participating in the study. *Source* Created by the authors

In this analysis, we used data from the qualitative component of two hospitals (Hosp 04 and Hosp 05) randomly chosen among those participating in M1 and M2 (Fig. 1). One of the hospitals is located in the Southeast region of the country, in a capital city with about 300 thousand inhabitants and Human Development Index (HDI) of 0.845 (Brazil HDI 0.699 in 2010), while the second is located in a city in the South region, with about 600 thousand inhabitants and HDI of 0.809 [16]. Both regions are the most developed in the country and have the highest proportions of health insurance coverage and high cesarean rates. In hospital 04, the cesarean rate presented to ANS was 65.5% in 2017; 63.18% in 2018; and 60.9% in 2019. In hospital 05, these rates were 70.56%, 72.32% and 72.32%, respectively.

**Fig. 2:**
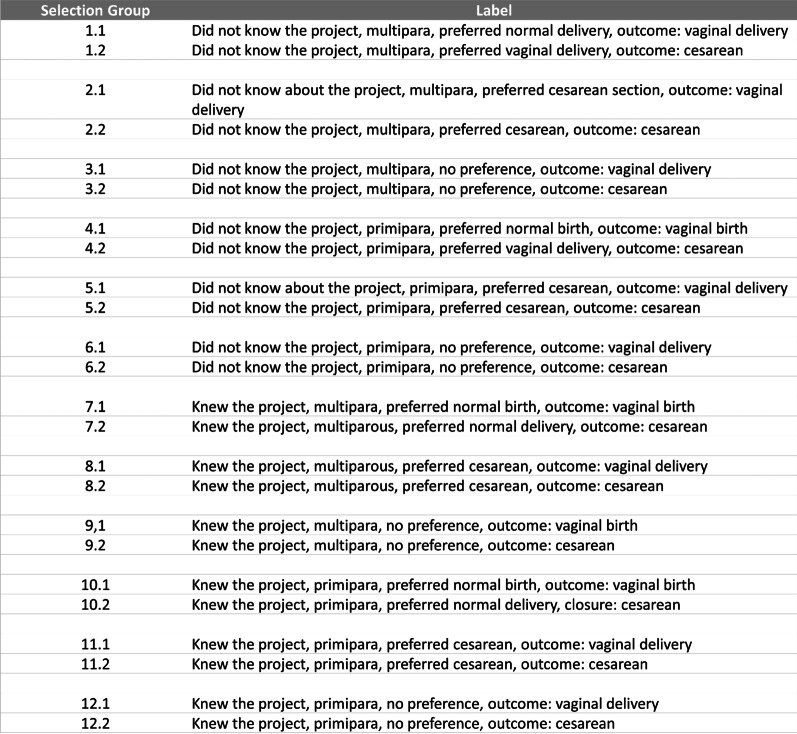
Classification of women for the qualitative stage—in-depth interview through telephone calls. *Source* Created by the authors

In both hospitals, we only used data from telephonic interviews with women. Based on data collected in the quantitative stage, 804 women from M1 and 729 from M2 were classified into 24 groups considering the variables “knowledge about the project” (yes or no), “parity” (primipara or multipara), “preference for the type of delivery” (vaginal, cesarean section, no preference), and “type of delivery” (vaginal or cesarean section) (Fig. 2). A minimum representation of one woman in each classified group was sought through non-probabilistic convenience sampling [17]. This sampling strategy included women of all groups and allowed reaching the minimum number of 20–30 interviews recommended by authors when conducting qualitative investigation [18].

Women were contacted via WhatsApp® to (re) present the research, create an initial bond, and invite them to the audio interview. In cases of acceptance, there was a telephone contact at the most appropriate time for the woman. The final sample included 102 women (M1: Hosp 04—36 respondents, Hosp 05—19 respondents/M2: Hosp 04—23 respondents, Hosp 05—24 respondents). The women’s non-response by message or phone call, in three attempts at different times and days, was considered a refusal (n = 18).

The interviewers who collected the data were trained in two ways: in person and remotely. The training guidelines included reading the instrument, presenting the procedures for conducting the interviews, field observations and the importance of records.

The 04 (four) interviewers were women with academic training in Nursing, Obstetrics, and History. The historian was a public health researcher, while the nurses and midwife were women’s health researchers; all had previous experience with data collection. Their previous experience in women’s health research or care practice was the main reason for participating in the study, and they were presented to respondents as such.

We used a semi-structured instrument [4], which included axes related to prenatal care, the choice of the maternity hospital and visit to its facilities before admission for delivery care, expectations, and experiences about childbirth, and the PPA strategy. A pilot test was carried out with a woman, in which the script was tested, and adjustments were made. This woman who participated in the pilot test was not included in the analysis of this paper.

The interviews were recorded with the verbal authorization of the interviewees, who had already signed a Free Informed Consent Form (FICF) for all the stages of the research at the time of their hospital admission for labor and delivery care. The interviews were carried with a duration that varied according to the subjectivity of each interviewee (approximately 30 min). The audio transcriptions were made by an independent professional and reviewed by type sampling by the research team. There was no need to repeat interviews and field notes.

**Fig. 3:**
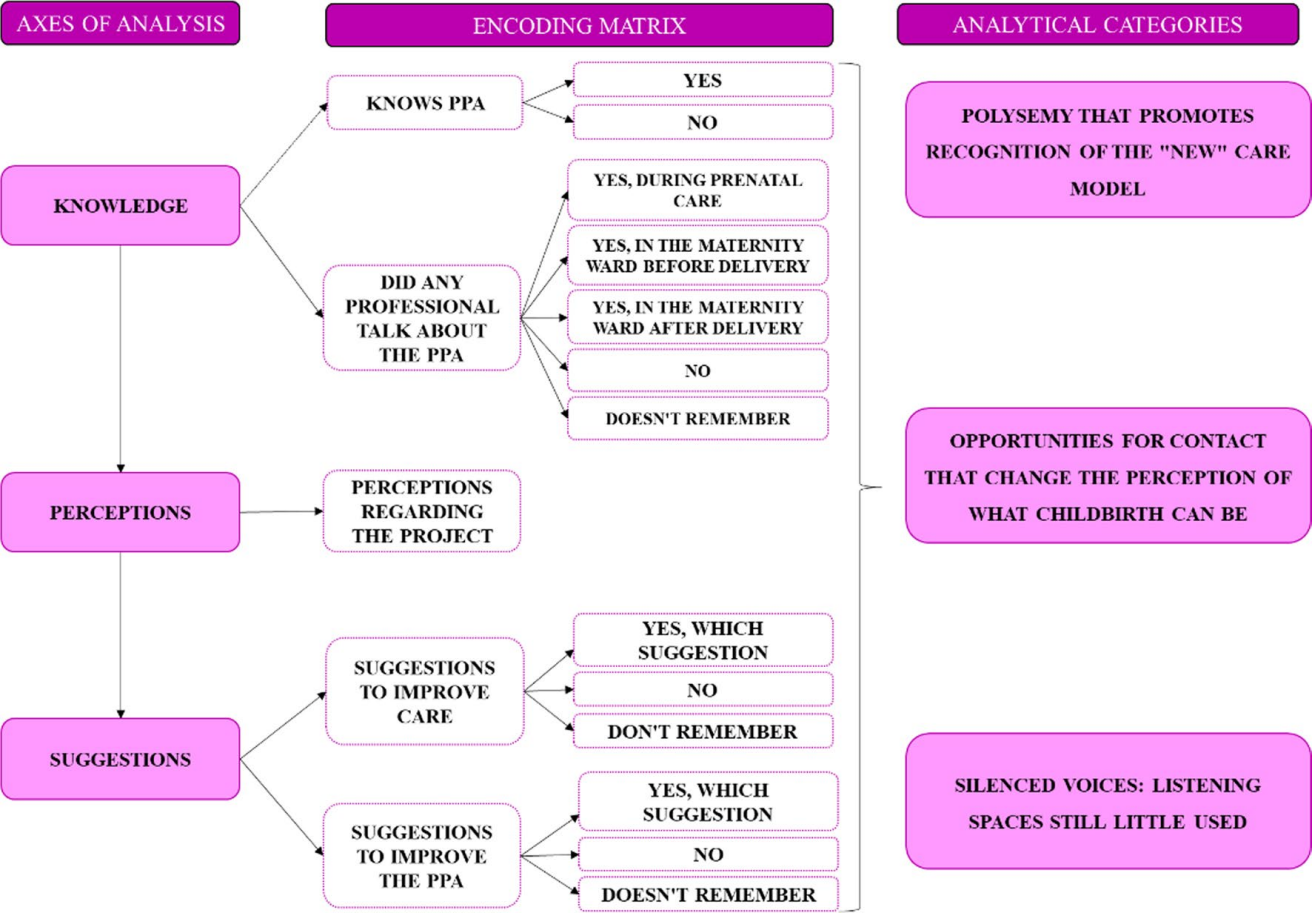
Axes of analysis and analytical categories. *Source* Created by the authors

Data were subjected to Thematic Content Analysis, according to Uwe Flick [19], using MaxQda software, 2021.1 version. The interviews were imported into the software, organized and encrypted according to their respective maternity hospital, research moment (M1 and M2), unique numbering of the woman, and route of delivery. The encryption used was identified only in the research dictionary, for greater security in relation to the anonymity of the interviewees. The open categorization was then carried out, generating broad segments, which were later refined, and a list of codes was generated from them, a step called axial coding. From this list of codes, an inductive association was made to create categories [19]. These initial categories were based on the research instrument. By identifying trends and crosscutting themes in the analysis process, issues were selected to be prioritized in the final analysis that we present here (Fig. 3).

Regarding the research quality criteria, the interviewers were trained, and the interview script was tested [4]. Although there was no feedback from the participants regarding the findings for the interpretation of the data, the interviewees’ speeches and their respective coding were validated by members of the research group, in which there were also reflexive co-participations on the interpretive procedures of the entire analytical phase [19]. To minimize possible identification, all citations in this paper indicate an encryption key. We used the consolidated criteria for reporting qualitative research (COREQ) [20] as a methodological guide.

## Results

The group of interviewees comprises mostly white skin color women (70%), with higher education (72% post-graduation), with paid work (85%), aged between 20–34 (71%) and ≥ 35 years (27%), and married or living with a partner (96%), a sociodemographic profile similar to that observed in a previous study in Brazilian private maternity hospitals [21]. The similar distribution between primiparous and multiparous women as well as the type of delivery were part of the selection criteria and, therefore, characterize this group and does not necessarily reflect characteristics of the population of women users of the private sector in Brazil (Table 1).

**Table 1:** Social-demographic and obstetric characteristics of participants according to study stage and maternity, Brazil, 2017–2018

	Moment 1 (implementation) (n = 55)		Moment 2 (sustainability) (n = 47)	
	Hospital 04 (n = 36)	Hospital 05 (n = 19)	Hospital 04 (n = 23)	Hospital 05 (N = 24)
	n (%)	n (%)	n (%)	n (%)
Parity				
Primiparous	19 (52.8)	9 (47.4)	13 (56.5)	13 (54.2)
Multiparous	17 (47.2)	10 (52.6)	10 (43.5)	11 (45.8)
Type of delivery				
Cesarean section	17 (47.2)	8 (42.1)	10 (43.5)	11 (45.8)
Vaginal birth (includes forceps e vacuum extractor)	19 (52.8)	11 (57.9)	13 (56.5)	13 (54.2)

The analysis of the axes Knowledge, Perceptions and Suggestions produced three analytical categories that will be presented next.

### Polysemy that promotes recognition of the “new” care model

In this category we identified that the PPA was unknown to most women before delivery. When citing as a “project”, the women brought the terms humanized childbirth, appropriate childbirth, and even “research” as part of the same set of actions that were pointing to changes in what they associated with vaginal birth and interventions that promote comfort during childbirth.

“I had no knowledge of the project, per se. […] As I really wanted it to be a normal birth, I wanted the opportunity to have a bath, for example, hot water on the back and so on. And then she said, ‘there they have the humanized delivery room. There is the Pilates ball, there is the shower, which you can use, there are some things that you can use. You can listen to music, you can bring a doula to accompany you, and your companion’. All these things. So, that’s what made my decision. I didn’t know the Project itself. I got to know a little bit about it when the first woman came to ask questions after my baby was born. It was then that I came to understand that it was a project and so on”. (S1_01_CS)

By confusing the term, it can be seen that the women participating in the research also confused the “project” and research teams, or even dealt indistinctly with the contacts made by the health plan operator or the maternity hospital, and even the research team.

“No, I don’t remember that name. She told me it was a research that was being conducted, explained to me about the research, but I don’t remember her saying it was a project and not the name of the project”. (S2_01_VB)

“Before, during pregnancy they called to do a follow-up by telephone, the people from [name of health plan operator] and talked about the Adequate Childbirth and talked about the lectures”. (S2_02_VB)

There were no major changes regarding knowledge about the PPA before delivery between M1 and M2. However, even though women did not know about the PPA, some sought the hospital because they identified that a differentiated care was offered there, a “new” model of childbirth care, a humanized care, especially among women who had vaginal deliveries.

“You just have to arrive at the hospital, say that you want a humanized birth and obviously, I chose a hospital that already had this whole team for this. So I loved it, because there, the doctor who attended me [on duty team], I arrived and my waters had already broken, I told her that I wanted a vaginal birth, but I did not say: ah, I want a humanized birth. I said: Doctor, do you think it’s possible to have a vaginal delivery? She said: Yes, it can be, and it’s better if it’s vaginal. So, that’s it, I want to have a vaginal birth. I did not even say I wanted humanized and I was very well treated”. (S1_01_VB)

“I already knew the hospital, some friends had already had children there and when they had them I went there to visit and everything, I was already preparing to get pregnant, so I already knew how it was at the hospital. The only thing I went afterward for information was a doctor to do the prenatal care, but regarding the hospital, since the beginning, before I got pregnant, I already knew that if I got pregnant, I wanted to have the child there”. (S1_01_VB)

When identifying maternity hospitals that promoted vaginal births, women mixed nuances of both the physical structure and the maternity team, especially about being well taken care of. However, the physical structure also included the availability of a Neonatal Intensive Care Unit, with maternity hospitals with this service perceived as a safer place to have their babies.

The recognition of the PPA, even months after delivery—which happened to some women during the qualitative interview—made them realize that it might have been interesting to have identified the PPA before, so as not to “miss opportunities” made available by the project. Thus, if all women knew about it and were informed beforehand, it could result in a better birth experience. This understanding of the importance of the project for changes in childbirth care was favored by the participation in the “Healthy Birth” research, which contributed greatly to “explain” the PPA to the women.

“What I see, as a movement [of maternity ward 04], especially, which I think is very cool, is this issue of humanization. Now, this care for the mother, this issue of obstetric violence, that this does not happen, the interventions, the unnecessary interventions, I believe it has to do with this project. That it is an initiative of this Project, really, this humanization of the relationships at the time of delivery”. (S1_01_VB)

“They talked [about the PPA in the maternity course]. They even explained that the [health plan operator], which is where we delivered our babies, had a much higher percentage of cesarean sections, before they entered this program. “It didn’t [influence the choice of maternity], because I didn’t know before. In this case, I only learned about it during the course for pregnant women”. (S2_02_CS)

“Not in the maternity hospital, but I have heard [about the PPA] in another environment, I won’t remember where I first heard about it”. (S2_01_CS)

Three aspects should be highlighted. The first is the recognition of the PPA, especially in M2, when women acknowledged the changes proposed by the project with details of labor and delivery care received. Women felt safer and more comfortable to give birth in a maternity hospital when they recognized that the service had professionals focused on the wellbeing of the pregnant woman and the baby, in addition to the usage of best practices and encouragement of vaginal childbirth. This reinforces the importance of disseminating information about PPA before admission to delivery care. The information provided during pregnancy should also favor the reduction of cesarean rates, since it demystifies aspects related to vaginal delivery and favors a more appropriate choice of the delivery route. The recognition by women of the relationship between PPA and changes in the model of childbirth care suggest that hospitals are implementing activities foreseen in the project’s theoretical model (reorganization of care, promotion of vaginal delivery, among others).

The second aspect is related to a specific group of women, composed of employees of the maternity hospital and that were also users of the services at the time of their deliveries. By knowing the PPA more closely, they had a greater perception of its benefits. Because they work at the health unit and observe the day-to-day implementation of the project, there is a prior knowledge that reaches their perception about childbirth and the proposed model of care; thus, when going through the experience of pregnancy, they assume the desire for vaginal delivery and assume the maternity hospital’s commitment to achieve the objectives of the PPA.

“Yes, Adequate Childbirth is a project that aims to reduce elective cesarean sections and also to avoid having so many cesarean sections. I understood well how the project works”. (S1_02_VB)

The third aspect is the implementation of guided visits to the maternity ward before delivery time. These visits aim to increase women’s knowledge about the dependencies of the maternity hospital and about the practices that will be carried out during the hospitalization period, including those that facilitate childbirth. Women who received explanations about the PPA at that time, upon learning about the project and its objectives, reported that they felt more secure in having their children in the maternity service, as the focus of the practices would be the parturient and child’s well-being. In other words, not only the changes in the maternity environment are presented, but also the processes adopted in the “new” model of care.

### Opportunities for contact that change the perception of what childbirth can be

The participation in courses for pregnant women, promoted by the maternity hospital or by the health plan operators, and the visit to the maternity hospital during pregnancy, were mentioned as opportunities to get to know the PPA. A greater knowledge about the project was noticed among participants when they participated in these activities. The practices promoted by PPA were recognized in a positive way, as practices that “encourage vaginal birth”, as reported by one of the women (S1_01_VB):

“I will tell you what I understood. I may be mistaken, but what I understood is that this project, this research that you are doing, is to encourage women to have a humanized childbirth, a vaginal birth […]. I believe that was it. I thought the project was sensational, the research, the willingness of each professional who was there, the woman who attended me, […] asking, clarifying, talking about your educational project and I thought it was sensational, very good”. (S1_02_VB)

The groups for pregnant women are also important opportunities to exchange information about pregnancy, delivery and postpartum and to dispel myths about the childbirth. Therefore, when women refer to an “adequate childbirth”, it is not only the name of the project, but the knowledge that there are other possible ways of experiencing childbirth, especially for those women who did not consider a more active participation in childbirth.

“I found it very interesting that the hospital has this project, because first of all, by participating in the training it removes some of the doubts, sometimes the fear that we have, if we are first-time mothers and so on. There is a certain taboo, a certain fear. I became a mother at thirty-four and despite my age, I had no information about what happens at that time. If I go into labor, what do I do? Will the baby be born right away? So, I heard all this at the lecture”. (S2_02_CS)

“Yes [they talked about the PPA in the visit]. They talked about everything I had learned in the conversation circle, in the health plan [operator] and they talked about the importance of the golden hour, that my baby could stay with his mother after birth, that they did not give a bath in the first twenty-four hours of the baby’s life, that breastfeeding was joint, everything I had already heard in the conversation circle”. (S2_01_CS)

Women valued the opportunities to contact the hospital and the operator before admission for childbirth and the PPA was recognized as a disseminator of information about types of birth and women’s rights during labor and delivery. Although there are still few women participating in the groups, courses, and visits, the results indicate that the implementation of these activities is relevant to promote greater support for women in their parturition process. Even among women who have had their babies by cesarean section, the PPA was perceived as an agent that promotes the humanization of care. This humanization, in all its polysemy, includes not only practices related to the moment of delivery, but also the personalized approach to women. For women, an adequate birth is not restricted to a vaginal delivery but combines a set of values that can promote a more meaningful birth experience.

“Actually, this question (…) is personalized. Of wanting to listen to each person, what they have to add, the doubts of each person, very individualized”. (S1_01_CS)

It is worth noting that among women who did not remember the project or did not remember what they said about the PPA during the hospital interview, their birth experiences seem not to have been affected by the changes underway at the maternity hospital. This was unrelated to the type of delivery, as both women who had had vaginal birth and cesarean section reported having no memory of PPA.

It was during the M2 that opportunities for contact with the PPA gained relevance, when women reinforced that the project was important to understand the birth process and to alleviate their fears. Even when this knowledge has not affected their childbirth experience, they have changed their perspective on childbirth and the way they will share it with other women and family members.

“From what I remember of the day I went [to the course for pregnant women], they talked about this situation, about vaginal birth, and sometimes it’s not everything they talk about, because sometimes the person is scared, they don’t know what’s going to happen during the birth, the baby is going to pass through and then it’s like, am I going back or not. I found the information very interesting. They talked about everything. It broke taboos. Some information for people who, since it’s the first pregnancy, we don’t know. We have never experienced that.” (S2_02_CS)

It was also during M2, especially in hospital 5, that women were able to report in more detail the experience they had during childbirth. It is hypothesized that this narrative construction may be related to better experiences, which may also be an effect of the way the PPA is conducted by the maternity hospital.

### Silenced voices: listening spaces still little used

This category discusses the participation of women in the PPA. We identified that the postpartum contact made by the maternity hospitals aims to assess satisfaction with the service. In general, the suggestions that women give to the maternity hospitals are limited to the physical structure (shelves for souvenirs, parking, and availability of rooms) and the service (service by on duty physicians, for example).

“Then, I think someone came to ask me some questions, how it had been, like a short interview. They asked me and the other mother who was sharing the room with me. They asked me some questions, like how we had been treated, how the birth had been, if it was vaginal, if it was (…), but something very simple, very superficial”. (S1_01_CS)

“In fact, a suggestion we made, that has nothing to do with childbirth, that the doctor herself later asked for and now it seems they even adopted it, to have a shelf for us to put the souvenirs, food and drinks, such things like that, but I didn’t make any suggestion about childbirth, no”. (S2_01_CS)

The suggestions related to the availability of rooms for vaginal delivery were mainly part of the narratives of women from maternity hospital 04, who preferred a vaginal birth and knew about the existence of a room prepared for this type of delivery. The availability of only one room was signaled as a limitation and points out to the need for expansion by the maternity hospital.

“I think it is a trend, presently, vaginal birth. So much so that I remember that there, at the course for pregnant women that [Maternity 04] offers, they said they were even expanding it, because [Maternity 04] only has one vaginal delivery room. If I’m not mistaken, their idea was to increase this number of rooms, because the number of women seeking this type of delivery, natural or vaginal, or whatever is appropriate for her and the baby, right, start with vaginal and then end with a cesarean section, is increasing. So, I’ve had the experience, for example, when I arrived, the room was dirty. They had to clean it to receive me. So much so that my bag broke in the corridor. I have already had the experience of a friend of mine who arrived there, there was already someone in the vaginal delivery room and she had the baby in the room. So, I think the hospital has to have this alternative, yes”. (S1_01_VB)

“I think the thing that had the most impact on my delivery was the fact that the hospital only had one delivery room that was occupied and then, if I could make a suggestion to a hospital is that, a hospital that has the amount of patients that it has, that they have two delivery rooms, at least. I think that the most negative impact on my delivery was that it was a vaginal delivery in a surgical center”. (S1_01_VB)

Other suggestions for improvements in the physical structure included improving the spacing of the rooms and making them more comfortable, and having a parking lot available, as this would be a cause for concern before delivery.

“I remember I said, or rather, that we had to have the birth we wanted, I remember I said that. I complained (…). Actually, the hospital didn’t have its own parking lot, we had to leave it on the street. But I think I said it badly enough, because I was very angry. She [the prenatal doctor] took my hand, ‘calm down, calm down, it’s not your fault’. I almost killed my daughter”. (S2_02_CS)

The suggestions for improvement in the maternity teams were present in both maternity hospitals. Health professionals should give more attention and have more empathy, humanity, and be more welcoming during hospital admission and during labor and delivery care. Women also expressed concerns about the care provided by different on-duty teams, a change implemented in the maternity hospital after joining the PPA.

“They asked and that’s what I said. The issue of professional training, because the structure is cool, but if the professional is not well trained, it’s no use. The structure will collapse, I think. Even more for those who go for vaginal birth, who don’t use this structure so much”. (S1_01_VB)

“The doctor who was in the emergency room when I arrived, was horrible, but I only had the baby when he changed shifts and the doctor who came on duty, gee, she was wonderful”. (S2_02_VB)

It is noteworthy that in one of the maternity hospitals, women who had cesarean sections did not make any suggestions regarding the team that attends the birth. It should be considered that women might see limitations in intervening or suggesting changes in this context of cesarean section practice because they perceive it as “standardized” for being surgical, and therefore, they are not allowed to participate in proposing changes in the model of care.

Overall, almost half of the women did not make suggestions or do not remember the suggestions they made to improve maternity care when contacted by the maternity hospital staff. The women showed a lack of confidence in the contact made, as if it was not worth making suggestions beyond those related to physical structure and professional team. In other words, the women did not perceive this contact as a relevant listening space and did not feel any bond that would give relevance to their voices. However, women valued the opportunity to be heard during the research interviews, even months after delivery:

“I think it is very good to have someone to talk to, regardless of the connection with the hospital. I think it’s very good, because no professional at the hospital is willing to listen. It seems that they don’t. So, you can have (…) all right, there may be people there who had everything wonderful, but there are many who didn’t, and then, you have an ear to (…) not just to listen to your disappointments, because this is what friends do, but someone who is going to take it in stride to improve. This I find very interesting”. (S1_01_VB)

“They didn’t do a survey with me after the birth. A research that the woman had a clipboard, writing down, but I think it is interesting that the information has to come before. The health plan, I was completely unassisted. I think that there could be, like, a call, a ‘come here’, that I think they can find out by the amount of consultations, or else that the person should sign up: I am pregnant; I would like to participate in a program. Even to help in the decision, even”. (S1_01_CS)

When women feel that they are listened to, they feel part of the improvement process. Therefore, the increase in listening channels, a still small and non-systematized practice in the maternity hospitals studied, would increase the participation of women through their suggestions and contributions, including them as allies in the improvement of the project.

“I remember that at the time, I even said that I was very satisfied with my cesarean section and so on. Nowadays, I think I would wait a little longer, to try an induction, or try a vaginal birth”. (S1_02_CS)

“I thought it was interesting. Valid that stays for other people, right?” (S1_01_VB)

Using data from M1 and M2, we hypothesized that these listening channels had somehow improved. However, what we were able to identify is that women’s voices are still silenced in the improvement of the PPA. Their participation in the survey shows that they would strive to propose other types of suggestions, mainly because they hope that this will affect the experiences of other women.

## Discussion

In Brazil, the debate about the childbirth care model is polarized between medicalized and humanized childbirth [22]. However, “humanized childbirth” is a polysemic expression [23] that can be interpreted in different ways. This context leads women to confuse the terms “adequate birth” and “humanized birth” or to use them indistinctly. Not all women identified the PPA as a project, but many recognized that new practices were underway and even that there was a “new” model of care. This suggests that the lack of recognition of the PPA among the research participants are more related to the limited knowledge of the broader proposal of the project than to the lack of knowledge that changes are happening in the model of childbirth care in the maternity hospitals where they had their children.

Women’s participation in care decisions related to childbirth is recognized as fundamental for providing better childbirth experiences for women and their families [24–27]. The most recent World Health Organization guideline for intrapartum care focus on recommendations for a positive birth experience, which means that women should be the primary focus of care [28]. Women’s participation in pre- and post-partum processes can help to identify them as key participants in the quality improvement process implemented by PPA, or in other initiatives adopted by other maternity hospitals.

Information is a key point for participation. Some studies have pointed out the use of cell phones as a positive tool for disseminating information during the pregnancy-puerperal cycle [29, 30]. This strategy was mentioned by some women, but it was not clear whether the information was provided by the health plan operator, by the maternity hospital, or even through an app used by the women. Participation in prenatal group was valued by women and is an effective non-clinical intervention to reduce CS [31]. However, less than half of the women participated in antenatal groups or visited the maternity hospital before delivery [32].

These contacts before childbirth can contribute to the objective of increasing women’s participation in decisions related to the model of care they will receive in the maternity hospital, while contacts after childbirth can provide support during the puerperal period, reinforcing the woman as the center of care. However, postpartum contacts can also be an important source of feedback of the care received, transforming women into agents of the processes of change underway in the maternity hospitals, with the aim of promoting positive childbirth experiences [28, 33].

There are still gaps in knowledge about how to implement and measure woman-centered care [27]. In the “Healthy Birth” study, qualitative interviews were the last contact of the study with the participants, who positively evaluated the multiple contacts with the research team and the opportunities to be listened. The women reported that these contacts provided them with the opportunity to talk about their childbirth and reflect on the childbirth care received in the maternity hospital. They also perceived that their contributions/suggestions could have positive effects on the birth experience of other women. Women expressed expectations of a relationship with the maternity that is not limited to the moment of childbirth. However, no contact opportunities were mentioned in relation to the PPA, suggesting that women are not yet included as agents of change in this quality improvement project, as their voices are not represented in related processes.

The participation of women and families is one of the components of the theoretical model of the PPA and seeks to expand the “empowerment of women and families so they actively participate in the entire process of pregnancy, birth and postpartum care” [4, 6]. However, this component had the lowest degree of implementation during the first phase of the PPA [34]. It is possible that the implementation of the quality improvement project in the maternity hospitals prioritized other strategies—such as changes in the environment, restructuring of professional childcare teams, among others—leaving behind activities aimed at women and their families.

In Brazil, the assessment of women’s satisfaction with the physical structure and the availability of complementary services is already in place in many private maternity hospitals [35]. However, it is necessary to expand women’s listening mechanisms, promote interactive communication channels before, during and after childbirth, and involve them in decisions related to maternity and intrapartum care models, as pointed out by studies [25, 26, 28, 36]. The strategies and channels used by the “Healthy Birth” study, such as telephone contacts after childbirth, could be used and improved by maternity hospitals. A more active participation of women could also be promoted through the implementation of patient councils and the strengthening of ombudsman actions.

Changes in childbirth care models are complex and depend on public health policies, wide dissemination of information and on the continuing education of professionals involved in care to promote a respectful childbirth [37, 38]. In Brazil, there has been synergy between the social movements and proposals for change in childbirth care. The PPA itself is the result of a complaint filed with the Public Ministry by a group of women from the state of São Paulo.

However, this cannot be a unilateral movement made by women. Women should be involved in the implementation and sustainability phases of quality improvement projects, such as the PPA, or other initiatives to change the model of childbirth care [36]. Various initiatives can be used to listen to women and make them the center of care. However, the results of this study show that these initiatives are still fragile and that maternity hospitals need to make an active move to seek the voice of women.

The “Healthy Birth” study is the first research to evaluate a national quality improvement project to reduce unnecessary CSs and improve the model of childbirth care in private maternity hospitals. The study used mixed-methods of research, primarily quantitative, with qualitative components integrated into the data collection and data analysis [4], which allowed the selection and inclusion of women with varied characteristics in the qualitative analysis. Data were collected through a telephone interview that facilitated the inclusion of women residing in different regions of the country. Different procedures were adopted by the research team to ensure the quality of interviews, such as training the interviewers, testing the interview script, and validating the interpretation of data.

However, this study has some limitations. Although the study is part of a mixed methods project, this article only uses data from the qualitative approach. Mixed methods can improve future analyses. In this analysis, we only included women from two maternity hospitals located in the Southern region of the country, the most socioeconomically developed region. Brazil is a continental country and different results would be observed in maternity hospitals located in less developed regions, especially if participation in childbirth care was not perceived as a woman’s right. The assessment of women’s opportunities of contact to participate in the process of improving quality childbirth may have been affected by the study, as the research team made many contacts to listen to women after delivery. At each contact, women were informed that this contact was part of a research study, but some women may have misinterpreted the research contact as a maternity contact. Therefore, contact opportunities could have been even less frequent.

## Conclusion and recommendations

The participation of women in the processes of improving childbirth care is relevant and necessary. However, women’s participation is still incipient in two hospitals that are implementing a quality improvement project that has increased women participation as a one of its main components.

Women’s voices, when listened to, can be an important driver of change, especially if a positive birth experience is the primary focus of care. With this study, we raise the question: How is childbirth care organized without listening to women?

It is up to maternity hospitals to create or improve listening channels that incorporate the voices and demands of women before, during and after childbirth, in order to assess their experience and obtain suggestions that can contribute to their own future experience and that of other women.

## Data Availability

The datasets used during the current study are available from the corresponding author on reasonable request.

## Abbreviations

ANS: National Supplementary Health Agency
CS: Cesarean section
VB: Vaginal birth
PPA: Adequate Childbirth Project (Projeto Parto Adequado)
WHO: World Health Organization
